# Plant Growth-Promoting Rhizobacteria and Biochar as Drought Defense Tools: A Comprehensive Review of Mechanisms and Future Directions

**DOI:** 10.3390/cimb47121040

**Published:** 2025-12-12

**Authors:** Faezeh Parastesh, Behnam Asgari Lajayer, Bernard Dell

**Affiliations:** 1 Department of Soil Science, University of Manitoba, Winnipeg, MB R3T 2N2, Canada; parastee@myumanitoba.ca; 2 Department of Engineering, Faculty of Agriculture, Dalhousie University, 39 Cox Road, P.O. Box 550, Truro, NS B2N 5E3, Canada; 3 Centre for Crop and Food Innovation, Murdoch University, Murdoch 6150, Australia; b.dell@murdoch.edu.au

**Keywords:** ACC deaminase, char sphere, climate resilient farming, microbial colonization, phytohormone crosstalk, soil-water retention

## Abstract

Drought stress, exacerbated by climate change, is a serious threat to global food security. This review examines the synergistic potential of plant growth-promoting rhizobacteria (PGPR) and biochar as a sustainable strategy for enhancing crop drought resilience. Biochar’s porous structure creates a protective “charosphere” microhabitat, enhancing PGPR colonization and survival. This partnership, in turn, induces multifaceted plant responses through: (1) the modulation of key phytohormones, including abscisic acid (ABA), ethylene (via 1-aminocyclopropane-1-carboxylate (ACC) deaminase activity), and auxins; (2) improved nutrient solubilization and uptake; and (3) the activation of robust antioxidant defense systems. These physiological benefits are orchestrated by a profound reprogramming of the plant transcriptome, which shifts the plant’s expression profile from a stressed to a resilient state by upregulating key genes (e.g., Dehydration-Responsive Element-Binding protein (DREB), Light-Harvesting Chlorophyll B-binding protein (LHCB), Plasma membrane Intrinsic Proteins (PIPs)) and downregulating stress-senescence markers. To realize a climate-resilient farming future, research must be strategically directed toward customizing biochar–PGPR combinations, validating their long-term performance in agronomic environments, and uncovering the molecular bases of their action.

## 1. Introduction

The main challenge for 21st-century agriculture is to meet the escalating food demands of a growing global population while simultaneously mitigating and adapting to the detrimental effects of climate change. This challenge is intensified by the increasing frequency and severity of abiotic stresses—primarily drought, salinity, and temperature extremes—which severely impair global plant growth and crop productivity [1,2]. Drought stress is one of the most significant environmental stresses for crop production [3]. Drought is characterized by an insufficiency of precipitation that significantly reduces soil moisture levels and changes the soil–plant–atmosphere structure, causing major physiological and biochemical disruptions in plants and agricultural production. Climate changes are expected to cause intense and frequent droughts, impacting plant growth in approximately 50% of agricultural lands by 2050 [4]. This stress has already impacted plant development on one-third of the world’s agricultural area [5,6].

In response to drought stress, plants alter their morphology, enhance water use efficiency, and activate protective systems to scavenge reactive oxygen species (ROS). While plant breeding has developed drought-tolerant crops, this approach is often time-consuming, expensive, and can yield unsatisfactory results due to the complexity of the trait [7]. Therefore, research into inputs and methods that can enhance crop productivity and plant health under drought stress is important for food security and sustainable farming [8]. One of these strategies is applying different soil amendments. Soil amendments are materials and organisms that are applied to the soil to improve its ability to promote plant development and survival. Compost, ferrous sludge, animal slurry, green manure, biochar, microorganism, liquid manure, and other organic soil additions have all been investigated as methods to boost soil fertility and crop productivity. Soil organic amendments can improve soil texture, increase soil fertility, maintain soil health over time, and, most importantly, increase crop yields.

Rhizobacteria are different types of bacteria whose growth is strongly influenced by root exudate and exchange, making them the dominant group of microorganisms in the rhizosphere. Within the rhizobacteria, a group known as plant growth-promoting rhizobacteria (PGPR) provides beneficial impacts on plant growth and yield. PGPR can help plants through multiple mechanisms, such as (1) nutrient acquisition via biological nitrogen fixation (BNF) and the solubilization of minerals like phosphorus (P), potassium (K), and zinc (Zn); (2) phytohormone modulation by producing growth promoters (e.g., auxins, cytokinins) and regulating stress hormones like ethylene; and (3) the production of siderophores to improve iron uptake and inhibit pathogen growth [9]. PGPR application has proven to be a promising and sustainable alternative to chemical fertilizers and pesticide application for increasing crop yield under drought stress [8]. PGPR can colonize plant roots, increasing soil fertility, promoting plant growth and development, and enhancing the yield of crops. A wide range of rhizobacterial genera have been studied for their potential to enhance plant growth and yield under drought stress, including *Alcaligenes*, *Arthrobacter*, *Azospirillum*, *Azotobacter*, *Bacillus*, *Burkholderia*, *Enterobacter*, *Klebsiella*, *Pseudomonas*, and *Serratia* [10].

Another amendment for increasing plant tolerance to drought stress is biochar, which is a carbon-rich substance produced by pyrolyzing biomass under limited oxygen. Biochar application can improve soil fertility, carbon sequestration, and immobilize both organic and inorganic substances [11,12]. Biochar can improve soil pH, soil structure, cation exchange capacity (CEC), and water holding capacity (WHC). It has also been used to alleviate environmental stress in plants from salinity, drought, and heavy metals, etc. [11,13].

The use of biochar combined with PGPR is a promising strategy for enhancing plant growth and stress tolerance [14]. Despite widespread recognition of the advantages of PGPR and biochar for alleviating plant drought stress, their synergistic mechanisms and combined potential are less understood. To fill this crucial gap, this paper first explains how drought affects plant physiology and the processes by which PGPR and biochar individually confer drought tolerance. We then critically examine research investigating the potential synergy between biochar and PGPR co-application to identify mechanisms of enhanced drought tolerance. Lastly, we identify future research directions to underpin the benefit of co-application of biochar and PGPR for sustainable agriculture in water-limited settings.

## 2. Methodology

This review was conducted to synthesize current knowledge on the synergistic mechanisms of PGPR and biochar in enhancing plant drought tolerance. To establish a clear cause-and-effect framework, the review first outlines the physiological impacts of drought before examining the mechanisms by which PGPR and biochar mitigate these effects. Literature search and analysis were performed following a systematic approach to ensure comprehensiveness and reproducibility. A systematic literature search was conducted using the Web of Science, Scopus, and PubMed databases for articles published between January 2005 and 2025. The search strategy utilized a combination of keywords and Boolean operators: (“drought stress” or “water deficit”) and (“plant growth promoting rhizobacteria” or PGPR) and (biochar) and (mechanism or physiology or synergy).

The initial search results were screened based on their titles and abstracts, followed by a full-text assessment for eligibility. Studies were included if they were original research articles that: (1) investigated the combined application of PGPR and biochar under drought conditions, and (2) examined physiological, biochemical, or molecular mechanisms of drought tolerance (e.g., phytohormone modulation, antioxidant defense, stress-responsive gene expression, or soil-water relations).

## 3. Impact of Drought on Plant Function and Survival

Plants under drought stress undergo a wide range of intricate physiological and molecular reactions that are intended to preserve function and conserve water. The first sign of drought stress in plants is a reduction in the rate of photosynthesis, which is mainly caused by reduced stomatal conductivity, which limits CO_2_ uptake and increases photorespiration. Photosynthesis can also be impacted by non-stomatal variables, such as limited or decreased activity of the enzyme RuBisCo, which lowers Photosystem II (PSII) efficiency and available CO_2_ in the chloroplast [15]. Plants can employ a variety of mechanisms to cope with drought stress, including the production of ROS, synthesis of stress hormones (ethylene or abscisic acid (ABA)), and modified root structure and morphology. Plants show both immediate or short-term and long-term reactions to drought stress [16]. Plants frequently respond in the short term by reducing carbon fixation, stomatal closure, growth inhibition, hydraulic changes, signal transfer, osmotic adjustment, and cell-drought signaling. The growth of the plant is typically not threatened by these reactions if the environmental situation and soil water rapidly return to normal. Long-term reactions include reduced transpiration area, metabolic adaptations, restricted shoot development, and kernel abortion [17]. Drought stress can limit plant growth and physiological characteristics through water balance changes, adjustments in osmotic pressure, plant hormones, and signals triggered by ROS [18,19]. Drought stress can damage plant cells, including photosynthetic pigments, cellular membranes, and osmolytes that regulate osmotic balance. To survive drought stress, plants rely on two crucial processes: physiological and developmental changes; and stress memory [20].

Drought-stressed plants adjust their stomatal conductance to achieve a balance between water conservation and carbon uptake. ABA signaling triggers ion fluxes in guard cells, which result in stomatal closure. This decreases transpiration water loss but also limits CO_2_ entry and photosynthesis [21]. Apart from stomatal regulation, drought significantly limits the growth of leaves. Water deficit causes smaller, thicker leaves with a lower specific leaf area, a result of decreased cell turgor and cell wall relaxation. These traits confer advantages such as decreased transpiration surface and increased water-use efficiency.

In addition to these quick reactions, plants can form a particular kind of “stress memory” that makes them more resistant to water deficit. Plants that have already experienced drought may exhibit physiological and epigenetic changes even after being rewatered, which may help them become more resilient to future stressors. For example, recurrent drought has been demonstrated to maintain reduced stomatal density, boost ABA production, and upregulate stress-related genes like RAB18 and NCED3, all of which promote stomatal regulation and water conservation [22]. These adaptive modifications allow primed plants to maintain leaf function, recover photosynthesis, and become more drought-resistant in general [22].

## 4. PGPR and Drought Tolerance

PGPR enhance plant drought tolerance through a multitude of mechanisms, including modulation of osmotic adjustment, phytohormone profiles, antioxidant defense systems, stomatal conductance, and nutrient acquisition. They also promote the production of bacterial volatile organic compounds (VOCs) and exopolysaccharides. Through these processes, PGPR can alter plants at the molecular, physiological, morphological, and biochemical levels [23].

**Figure 1:**
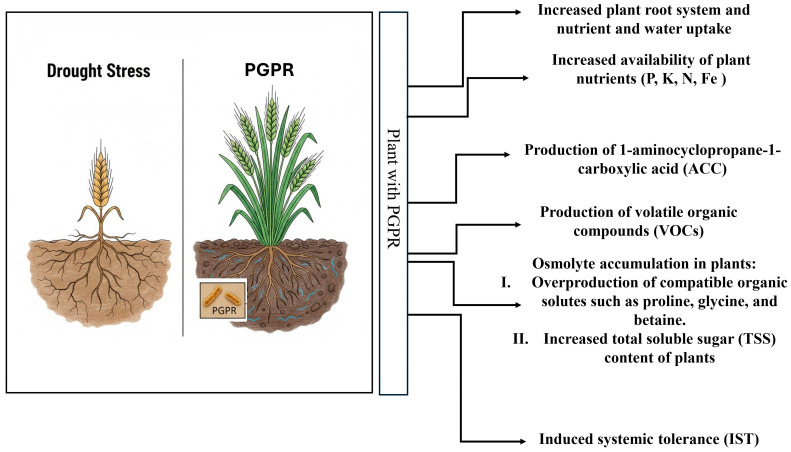
Key PGPR processes for reducing the physiological effects of drought stress in plants.

PGPR increase plant growth directly in both normal and stressed conditions. Direct mechanisms that promote plant health include BNF and the solubilization of minerals such as phosphorus, thereby improving plant nutrition. For instance, nitrogen-fixing PGPR such as *Paenibacillus polymyxa*, *Rahnella* sp., and *Serratia* sp. are able to increase the mineral nitrogen concentration in the soil solution. Additionally, PGPR can reduce nitrogen (N) leaching from soil by assimilating it into their own biomass, which improves nitrogen retention and availability for plants [24]. Also, many PGPR as well as fungi, such as arbuscular mycorrhizal fungi (AMF), can solubilize and release inorganic phosphate (Pi) in the soil solution, increasing Pi availability for plant uptake, leading to enhanced growth [25,26,27]. Figure 1 illustrates strategies used by PGPR to assist plants. Key mechanisms are detailed below.

### 4.1. Improved Nutrient Absorption

Drought stress primarily impairs the diffusion and mass flow of essential nutrients in the soil, thereby limiting their availability to plant roots. Inoculation with PGPR can mitigate these limitations and enhance nutrient acquisition. For example, barley (*Hordeum vulgare*) inoculated with *Pseudomonas fluorescens* SBW25 and *P. putida* KT2440 showed improved uptake of macro- and micronutrients under drought stress. Specifically, the uptake of calcium (Ca), magnesium (Mg), sulfur (S), K and P, and, as well as beneficial elements such as aluminum (Al) and silicon (Si) in PGPR-treated plants was increased [28]. The study also reported a significant increase in chloride (Cl^−^) content in the roots, stems, and leaves of PGPR-inoculated barley under drought stress which may contribute to osmotic adjustment. Moreover, PGPR applications were effective in enhancing the uptake of the essential micronutrients cobalt (Co), copper (Cu), iron (Fe), manganese (Mn), nickel (Ni), and Zn [28]. A synergistic approach to improving nutrient uptake, particularly under stress, involves co-inoculation with PGPR and AMF. For example, K uptake in white clover (*Trifolium repens*) increased by 2.2-fold and 3.5-fold in plants inoculated with a combination of AMF and *Bacillus thuringiensis* or AMF and *Pseudomonas putida*, respectively [29].

### 4.2. Alteration in Phytohormone Levels

PGPR inoculation can modulate many plant hormones thus improving drought resilience by enhancing root growth, water retention, and stress signaling networks [30]. Of particular importance is the role of PGPR in helping to reprogram root system architecture (RSA) that increases the absorptive root surface area, which is crucial for water and nutrient foraging under drought [31]. In general, PGPR can produce indole-3-acetic acid (IAA) [32], which enhances the development of adventitious and lateral roots. Plants release the IAA precursor L-tryptophan into the rhizosphere, where PGPR convert it into IAA that can be taken up by the plant [9]. Also, IAA can be synthesized by a variety of PGPR through other pathways [33]. These include: via indole-3-acetamide (IAM) in *Pseudomonas fluorescens* and *P. putida* [9]; via the conversion of tryptophan to indole-3-acetonitrile (IAN) in *Ensifer* and *Rhizobium* [33]; and via indole-3-pyruvic acid (IPA) in *Agrobacterium*, *Azospirillum*, *Bradyrhizobium*, *Enterobacter*, *Klebsiella*, *Pseudomonas*, and *Rhizobium*. In addition, *Azospirillum* is thought to utilize the tryptamine pathway, which converts tryptophan to tryptamine first, then to IAM. Lastly, a tryptophan-independent mechanism is present in some azospirilla and cyanobacteria.

Drought stress often suppresses the production of endogenous plant auxins. However, IAA-producing PGPR working in combination with endogenous plant IAA can enhance drought stress tolerance. A significant number of these IAA-producing strains also exhibit moderate to high tolerance to osmotic stress (simulated with polyethylene glycol), a key trait for surviving in drought-affected soils. This suggests that these bacteria are not only capable of producing the vital phytohormone but are also naturally adapted to the dry conditions in which their host plants struggle, making them effective partners in enhancing drought tolerance [34]. Inoculating tomato (*Solanum lycopersicum*) plants with a strain of *Azospirillum brasilense* capable of producing nitric oxide (a diffusible gas that functions as a signaling molecule in the production of IAA) increased adventitious root development and improved plant tolerance to drought stress [35]. In another study, inoculating beans (*Phaseolus vulgaris*) with *A. brasilense* during drought stress resulted in increased auxin production and enhanced root length of plants in comparison to plants that were not inoculated [1].

### 4.3. Emission of Volatile Organic Compounds

Bacterial VOCs are key infochemicals that facilitate plant growth and induce systemic resistance (ISR) against biotic and abiotic stresses. These low-molecular-weight (<300 g/mol), lipophilic metabolites can diffuse easily through the soil matrix, enabling plant–microbe communication over a distance. Among the first bacterial VOCs identified for plant growth promotion were 2,3-butanediol, acetoin, and 2-pentylfuran [36]. Prominent VOC-producing genera include *Arthrobacter*, *Bacillus*, *Pseudomonas*, *Serratia*, and *Stenotrophomonas*. Various other VOCs identified from soil microbes include alkanes (e.g., decane, dodecane), chlorinated compounds (e.g., 1-chlorooctadecane), and complex organics (e.g., 2-(benzyloxy) ethanamine) [37]. Particularly well-studied are the VOCs 2,3-butanediol and acetoin from *Bacillus* species, which can simultaneously inhibit fungal pathogens and enhance plant biomass [38].

### 4.4. Synthesis of Exopolysaccharides

The production of extracellular polymeric substances (EPSs) by PGPR is essential for promoting plant growth under drought because EPSs form hydrophilic biofilms which protect plant roots from desiccation [39]. Bacterial biofilms mostly comprise homo- and heteropolysaccharides, which adhere to the cell surface as a slime layer or capsule. PGPR under stress produce guanine cyclase, which leads to the creation of EPSs [40]. While the polysaccharide composition varies with PGPR taxa, glucose, galactose, and mannose are common monomers. These diverse polysaccharide components can hold up to 70 g of water per gram of polysaccharide, which leads to variation in water retention capacity among PGPR. During drought, EPSs can help maintain the rhizospheric moisture content.

The role of EPSs extends beyond direct water retention to actively improving the soil physical environment. The sticky, polymeric substances act as a binding agent that cements soil particles together into stable aggregates. This aggregation is vital for creating a porous soil structure that enhances water infiltration and protects against surface crusting, thereby increasing the plant-available water content in the root zone. Furthermore, this biofilm interface forms a protective “rhizosheath” that buffers roots from abiotic stress and concentrates cationic nutrients through chelation, enhancing their bioavailability for plant uptake during a period when drought limits nutrient diffusion in the soil solution [41]. Also, the cation-binding capacity of EPSs plays a critical role in mitigating salinity stress, which often co-occurs with drought. By binding sodium ions (Na^+^) in the soil, EPSs reduce Na^+^ uptake by plant roots, thereby helping to maintain ion homeostasis and protect essential processes like photosynthesis [42].

### 4.5. Osmotic Balance Adjustment

Under drought stress, PGPR can help plants in maintaining osmotic equilibrium by increasing the accumulation of suitable osmolytes [43]. Proline is one of the most critical osmolytes, whose significant accumulation in plant tissues during water deficit is a well-documented adaptive response. This is clearly observed in non-inoculated plants, such as in semi-leafless pea (*Pisum sativum*) cultivars, where progressive soil drought led to a uniform increase in proline (and other metabolites like malate and myo-inositol) across all shoot parts, with a pronounced translocation to the shoot tip to protect the youngest tissues [44]. The vital role of proline lies in its high hydration capacity, which allows it to bind to proteins, enhance their solubility, and prevent denaturation [13,45]. Furthermore, proline helps regulate cellular redox potential by neutralizing free radicals, thereby mitigating oxidative damage.

The potential of external applications to enhance this mechanism is significant. For instance, soaking chickpea (*Cicer arietinum*) seeds in a solution of proline, combined with antioxidants like ascorbic acid and glutathione, was shown to up-regulate the plant’s defense systems, leading to improved growth under drought stress [46]. This principle is directly harnessed by PGPR. The proline content of cucumber (*Cucumis sativus*) plants under drought stress increased fourfold upon inoculation with a consortium of *Bacillus cereus*, *B. subtilis*, and *Serratia* sp., demonstrating how PGPR can actively amplify this vital defense mechanism [47]. Beyond proline, PGPR can also influence the production of other protective osmolytes, including polyamines such as putrescine (Put), spermidine (Spd), and spermine (Spm). These polyamines are versatile compounds that not only help with osmotic adjustment but also act as antioxidants, directly scavenging ROS and stabilizing membranes and proteins, which collectively enhance a plant’s capacity to tolerate dehydration [48].

## 5. Drought Regulation by the Application of Biochar

There is considerable interest in the use of biochar as an organic amendment in sustainable agriculture [49,50]. Biochar is created by thermally breaking down organic materials using pyrolysis. The combination of stable and labile components in biochar makes it a very diverse carbon source. The main components of biochar include moisture, carbon, ash, and volatile material [51]. According to Tomczyk et al. [52], biochar contains a variety of mineral nutrients, including N, P, K, Ca, Mg, Zn, and others, depending on the source of organic matter and pyrolysis temperature. Once added to soil, nutrients are released into the soil solution as biochar gradually decomposes [24]. Biochar has a porous structure and a large surface area that can create an optimum habitat for soil microorganisms. The high levels of organic carbon in biochar enable it to enhance the physicochemical and biological attributes of soil, which in turn can improve its capacity to function as a soil conditioner [53]. Biochar can enhance the growth of crops and help improve soil and water quality when crops are under stress [54]. Several studies have shown that applying biochar to soil that is infertile and suffering from drought, along with fertilizer, can lead to higher crop yields [55].

Biochar can improve soil porosity, moisture retention, and water-use efficiency, which helps mitigate the detrimental impacts of drought on crop yield [55,56,57]. Also, biochar can decrease N losses and increase the soil’s capacity to hold water [58]. Many factors influence the efficacy of biochar, including the temperature at which pyrolysis takes place, the biochar’s age, soil type being amended, and the plant species. Because precipitates, including P, Ca, and Mg, develop when biochar is generated at high temperatures, the higher pH induced may lead to P deficiency in crops on alkaline soils.

Due to its porous structure, large surface area, and capacity to absorb microorganisms and organic compounds, biochar can offer a habitat for PGPR (i.e., colonization, reproduction, and growth) when used with PGPR inoculants. Additionally, biochar can protect PGPR from damaging organisms [59]. Biochar supplies energy and the necessary nutritional building blocks for the survival and growth of inoculants due to its abundance in carbon, or substrate, and critical nutrients [60].

Biochar can help maintain a good soil environment for plant development by increasing soil water content and diluting ion concentration under drought stress. Various studies have shown that biochar can enhance soil moisture. According to Boretti and Rosa [61], biochar also alters the physicochemical properties of soils, potentially increasing enzymatic activity and microbial biomass. The efficacy of biochar as a soil amendment is highly dependent on its physicochemical properties, which are determined by the feedstock and pyrolysis conditions. Biochar produced at high temperatures (>350 °C) typically exhibits a higher pH, greater CEC, and increased aromatic carbon content, enhancing its stability and making it a superior tool for long-term carbon sequestration. Conversely, biochar derived from low-temperature pyrolysis (<350 °C) often retains higher concentrations of readily available nutrients like N and P [62].

This ionic homeostasis provides substantial protection to cellular membranes against drought-induced damage. Research has documented that biochar enhances the concentration of unsaturated fatty acids within membrane structures, thereby improving membrane fluidity and structural integrity under drought stress conditions [63,64]. Furthermore, biochar upregulates the activity of major antioxidant enzymes, including superoxide dismutase (SOD), catalase (CAT), peroxidase (POD), glutathione reductase (GR), and ascorbate peroxidase (APX), which collectively scavenge ROS and protect cellular structures from oxidative damage [65,66,67]. Emerging evidence indicates that biochar can enhance cellular water transport mechanisms by upregulating aquaporin (AQP) gene expression, particularly plasma membrane intrinsic proteins (PIPs), facilitating improved water movement into roots and leaves and thereby enhancing leaf water content under drought conditions [63]. At the molecular level, studies confirm that biochar amendment can upregulate the expression of key AQP genes involved in water transport. This enhanced genetic regulation, combined with biochar’s improvement of soil water retention, collectively contributes to higher leaf water content in drought-stressed plants [63].

The application rate is another critical factor; while optimal doses enhance soil fertility, excessive application can be counterproductive, potentially locking up nutrients, altering soil pH, or negatively impacting beneficial soil biota like mycorrhizal fungi [68]. In summary, the unique value of biochar lies in its complex structure, characterized by a large surface area, porosity, and diverse functional groups (e.g., hydroxyl, carboxyl) that form on its surface. These properties collectively enhance soil’s physical structure by improving porosity and water retention, its chemical fertility by increasing nutrient retention, and its biological activity by providing a protective habitat and carbon source for beneficial microorganisms such as PGPR. Therefore, the strategic selection of biochar type and application rate is paramount to harnessing its full potential in improving soil health and building crop resilience against drought stress [68,69]. Furthermore, biochar application has been shown to improve key plant physiological attributes, including stomatal conductance, transpiration rate, water use efficiency, and chlorophyll content, all of which contribute to maintaining photosynthesis under stress [70,71,72].

**Figure 2:**
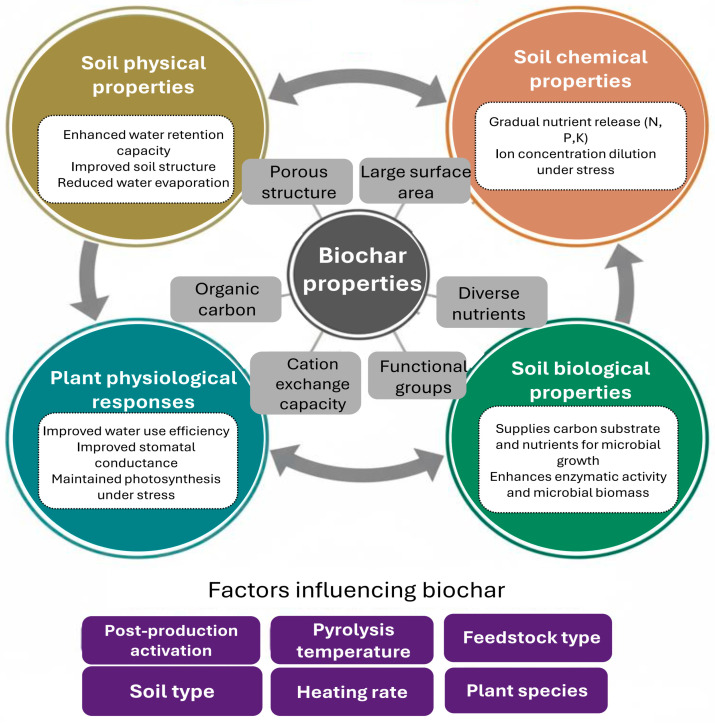
Key biochar mitigation strategies for reducing the effects of drought on plants.

Beyond its agronomic benefits, biochar is a multi-faceted tool for mitigating greenhouse gas (GHG) emissions and increasing the climate resilience of agricultural lands. A recent meta-analysis reported that biochar application can mitigate nitrous oxide (N_2_O) and methane (CH_4_) emissions from farm lands by about 13% and 7%, respectively [73]. This biochar ability, particularly for N_2_O, is driven by a significant change in soil microbial communities. Biochar is able to modify the functional gene pool, significantly increasing the abundance of the *nosZ* gene, which codes for the enzyme that can reduce N_2_O to N_2_ gas, thereby facilitating complete denitrification. As an example, application of coarse-sized wood biochar at 5 Mg ha^−1^ reduced N_2_O emissions in irrigated rice by nearly 90% without affecting yield [74]. Additionally, biochar has an effect on the carbon cycle. It can provide direct, long-term carbon sequestration through its highly recalcitrant structure, holding atmospheric carbon for centuries. Management practices can enhance its ability to suppress net soil CO_2_ emissions. A study of a wheat-corn rotation showed that biochar can mitigate CO_2_ compared to mineral fertilizer, primarily through reduced CH_4_ emissions and its superior capacity to adsorb CO_2_ in the soil [75]. Thus, in addition to improving plant productivity, biochar’s molecular-scale properties and longevity make it an effective means to increase the long-term soil C store and to reduce GHG emissions in food production [75]. Therefore, understanding the multifaceted benefits of biochar requires an appreciation of its core properties and how they interact with the soil–plant system. Figure 2 elucidates these relationships, detailing how biochar’s characteristics drive improvements in soil physical, chemical, and biological properties, as well as beneficial plant physiological responses. The figure also highlights the crucial factors that determine the efficacy of biochar.

## 6. Biochar as a Habitat for PGPR

The capacity of biochar to enhance soil quality and plant growth is profoundly interconnected with its function as a powerful modulator of the soil microbiome, operating through multiple integrated mechanisms that span physical, chemical, and biological domains [68]. As a porous carbonaceous material produced through pyrolysis of organic feedstocks, biochar possesses a complex internal architecture that fundamentally alters the soil environment. This architecture is characterized by a hierarchical pore network systematically categorized by material scientists into macropores (>50 nm), which serve as primary conduits for microbial habitation and hyphal growth; mesopores (2–50 nm), which function primarily in nutrient retention and surface area development; and micropores (<2 nm), which contribute substantially to the material’s immense specific surface area that can exceed 400 m^2^/g in biochar’s produced at higher temperatures [76]. This sophisticated porous infrastructure, combined with its demonstrated sorptive capacity for organic compounds, inorganic nutrients, and gases, creates a distinct ecological niche known as the “charosphere” that selectively enriches beneficial microorganisms through multiple mechanisms [77,78,79,80].

The colonization of this intricate network by soil microorganisms represents an active process mediated by specific physicochemical interactions rather than passive accumulation. Microbial attachment occurs through a combination of van der Waals forces, electrostatic attractions, and hydrophobic interactions, frequently facilitated by microbial EPSs and cationic bridges forming with oxygenated functional groups on the biochar surface [81]. The ionic character of the biochar surface, often derived from its inherent ash content containing cations including Ca, Mg, and Fe, plays a pivotal role in microbial adhesion through binding with anionic functional groups on microbial cell walls such as lipopolysaccharides [42,82,83]. This process is further enhanced by bacterial surface structures including flagellae, binding proteins, and the complexity of lipoproteins with multiple binding sites, where the cumulative effect of numerous weak binding forces from multiple functional groups on both biochar and microbial EPSs can generate binding energy comparable to covalent bonds, establishing remarkably stable associations [84,85,86].

The direct consequence of this engineered habitat is substantial transformation in soil microbial community structure, diversity, and metabolic functionality. A comprehensive meta-analysis encompassing 964 data from different studies published between 2007 and 2020 demonstrated that biochar application consistently and significantly increases soil microbial biomass carbon by 22% while enhancing the activity of crucial enzymes including urease (23%), alkaline phosphatase (25%), and dehydrogenase (20%), with no significant negative effects reported on any measured enzymatic parameters.

The stimulation of microbial life consistently leads to increased phylogenetic and functional diversity, a recognized hallmark of ecologically resilient soil ecosystems. Specifically, biochar amendments have been documented to increase the abundance of beneficial PGPR genera, including *Bacillus* and *Pseudomonas*, by up to 100%, while simultaneously suppressing populations of phytopathogenic fungi such as *Fusarium* and *Phytophthora* by up to 50% [87,88]. The efficacy of these microbial modifications demonstrates significant context-dependency, with biochar proving particularly effective in increasing microbial biomass in acidic, sandy-textured soils with low native organic carbon content, especially when the applied biochar is produced at pyrolysis temperatures between 350 and 550 °C and possesses a C/N ratio below 50 [89]. Furthermore, application rates significantly influence microbial community composition, with rates of 20,000 and 40,000 kg ha^−1^ shown to shift the community balance in favor of bacterial over fungal populations [90].

Biochar-mediated restructuring of the soil microbiome translates directly into enhanced plant physiological tolerance to abiotic stresses including drought and salinity through multiple interconnected pathways. Under drought stress conditions, which typically induce cytoplasmic dehydration and plasma membrane damage resulting in elevated lipid peroxidation and electrolyte leakage, biochar functions through several protective mechanisms [50]. As mentioned earlier, it significantly enhances the soil’s physical water-holding capacity through its porous structure and influence on soil aggregation, thereby increasing plant-available water. This hydrological improvement manifests in measurable enhancements in plant water status parameters, including increased relative water content (RWC), improved leaf water potential, and enhanced water use efficiency. This has been documented across multiple crop species including cabbage (Brassica oleracea), aubergine (Solanum melongena), chickpea, and wheat (Triticum aestivum) [90,91,92,93]. Application of biochar has been consistently demonstrated to reduce the concentration of malondialdehyde (MDA), a recognized biochemical marker of oxidative membrane damage, by enhancing the plant’s enzymatic and non-enzymatic antioxidant defense systems [94]. In barley, the combined application of chitosan and biochar improved drought tolerance by reducing lipid peroxidation and electrolyte leakage while enhancing membrane stability and relative water content [95]. Biochar, particularly at a 10% (*w*/*w*) application rate, played an essential role in alleviating drought stress in soybean (Glycine max). It positively modulated stomatal mechanisms, which in turn benefited physiological performance by increasing the net photosynthetic rate (121%), water use efficiency (88%), and biomass accumulation, thereby minimizing the deleterious effects of water deficit [96]. The charosphere’s ability to non-specifically enrich beneficial bacteria proves its value as a protective habitat and delivery mechanism for introduced PGPR consortia. Thus, an intentional modification of this ecological niche to optimize microbial activity and plant phenotypic outcomes is readily available through the co-application of PGPR and biochar.

## 7. Co-Application of Biochar and PGPR

The synergistic potential between biochar and specifically selected PGPR strains has been widely recommended for improving plant productivity across diverse agricultural systems. However, the majority of studies so far have been undertaken using containerized plants where the PGPR are often applied to the seed and the biochar is mixed into the soil or potting mix. These studies reveal that the combined application of biochar and PGPR generates superior outcomes compared to either component applied individually. In a study conducted by Wang et al. [47], PGPR strains of *Paenibacillus* and *Bacillus* combined with 2% biochar (millet straw) and N fertilizer significantly increased tomato yield. When *Bradyrhizobium japonicum* and *Pseudomonas putida* were co-inoculated with 3% biochar derived from maize, [97] observed a significant increase in plant growth indices. Compared to the treatment with 3% biochar alone, co-inoculation gave a 20% increase in seed germination, a 76% increase in root length, a 56% increase in root dry weight, a 41% increase in shoot length, and a 59% increase in shoot dry weight.

Similar findings have been observed in the smaller number of field trials that have been undertaken. For example, the combination of *Alcaligenes* sp. and 0.5 t ha^−1^ of maize biochar significantly increased plant growth compared to PGPR alone [86]. Plant height increased by 6%, grain yield by 14%, 1000-grain weight by 5%, shoot fresh biomass by 9%, and shoot dry matter by 12% as a result of the combination treatment [98]. Overall, when compared to treatments that just use PGPR or biochar, results show that co-inoculation with biochar significantly improves plant growth and production [99]. The mechanistic basis for these synergistic effects involves multiple interconnected pathways, including enhanced nutrient solubilization and availability.

**Figure 3:**
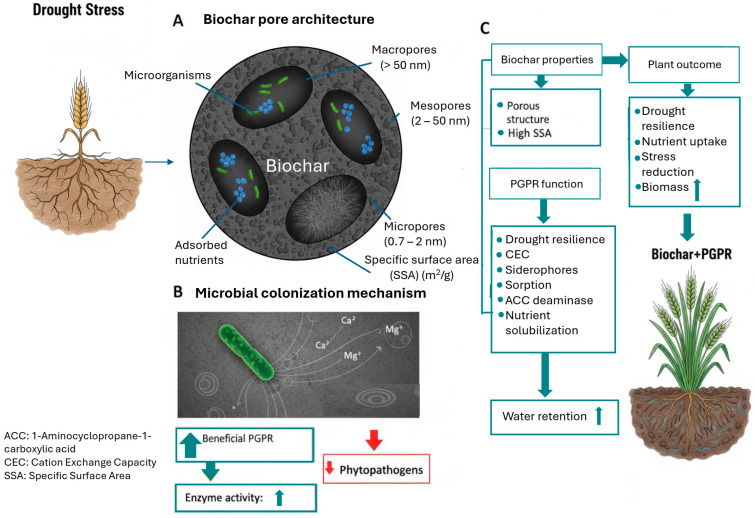
How biochar serves as a favorable habitat for PGPR and their roles in reducing drought stress in plants.

Figure 3 synthesizes the complex, multifaceted mechanisms by which the combined application of biochar and PGPR successfully mitigates drought stress in plants. The figure illustrates that this synergy is governed by both the physical properties of the biochar and the biological functions of the PGPR. Specifically, the highly porous structure of biochar (as detailed in section A) provides an essential macroporous network that increases the material’s SSA and CEC, allowing it to act as a microbial sanctuary and a nutrient reservoir. This physical habitat facilitates enhanced microbial colonization (section B), which in turn amplifies beneficial PGPR activities, such as the production of siderophores and the ACC deaminase enzyme. These concurrent processes ultimately lead to the desired plant outcomes (section C): superior water retention, improved nutrient uptake, significant stress reduction, and increased drought resilience, leading to greater biomass accumulation.

As mentioned earlier, PGPR are known for their ability to mitigate various environmental stresses, including drought, that hinder the growth and development of plants. As an illustration, the ability of PGPR to release exopolysaccharides under dry environments assists in the development of drought tolerance in plants [100]. Also, due to the high surface area-to-volume ratio of biochar and its high water-holding capacity, the addition of biochar can reduce drought stress in crops [23]. Synergies between PGPR and biochar have been discovered despite the fact that the mechanistic synergism between the two has not been extensively explored in these investigations. The capacity of biochar and PGPR to boost plant development and reduce stress is a result of the synergistic effects of direct and indirect processes. Higher soil organic carbon content and greater water retention capacities appear to be key drivers facilitating the biochar–PGPR dynamics.

Furthermore, biochar offers an optimum environment for microorganisms that defend against stress brought on by ethylene. The photosynthetic rate was considerably increased in a study [101] when *Pseudomonas aeruginosa* and two different types of biochar and *Bacillus amyloliquefaciens* were combined. Furthermore, the combination of biochar and drought-tolerant 1-Aminocyclopropane 1-carboxylic acid (ACC) deaminase-generating PGPR was more successful in reducing drought stress in wheat than single treatments. Among the PGPR strains tested, *Agrobacterium fabrum* and *Bacillus amyloliquefaciens* with biochar showed the greatest improvement in various aspects of wheat growth and production, such as gas exchange characteristics, nutrient concentration, photosynthetic pigments, and yield [10].

In another study, the increased surface area of biochar led to an increase in cation exchange sites in soil, ultimately improving the availability of nutrients [102]. This study found that when *L. adecarboxylata*, *A. fabrum*, *P. aeruginosa*, and *B. amyloliquefaciens* with biochar were applied, the electrolyte leakage in wheat leaves was reduced under both moderate and severe drought conditions compared to the control group. The decrease in electrolyte leakage may be attributed to the activity of ACC deaminase and improved availability of water and nutrients resulting from the combined application of PGPR and biochar. Lipid degradation by ethylene leads to the loss of membrane integrity, which allows ethylene to activate the chlorophyllase gene, causing damage to the chlorophyll molecules [103]. However, the addition of biochar and PGPR in combination resulted in a significant reduction in electrolyte leakage, potentially due to ACC deaminase activity, increased water availability, and improved nutrient uptake. Studies on the co-application of biochar and PGPRs under drought stress are summarized in Table 1.

Biochar functions as a keystone soil amendment that integrates physical, chemical, and biological mechanisms to comprehensively enhance plant resilience to environmental stresses. By creating a favorable habitat that enriches beneficial soil microbiome components and enhances key soil physicochemical properties, it initiates a cascade of effects that improve plant water relations, strengthen antioxidant capacity, and maintain membrane structural and functional integrity. The combination of biochar with selected PGPR creates particularly powerful synergies, as the biochar provides an ideal habitat for microbial colonization and protection while the PGPR enhance nutrient availability and directly stimulate plant defense mechanisms. This integrated approach represents a promising, sustainable strategy for enhancing crop productivity and system resilience in drought-prone agricultural systems, though optimal combinations require further investigation across different soil types, crop species, and environmental conditions.

## 8. Mechanisms of Enhanced Drought Tolerance in Plants Co-Supplied with Biochar and PGPR

The physiological and synergistic benefits of biochar and PGPR are ultimately orchestrated at the molecular level through a profound reprogramming of the plant’s transcriptome and regulatory networks. This reprogramming is not merely a suppression of stress pathways but a sophisticated re-allocation of resources, guided by specific hormonal and environmental cues [119]. Biochar’s primary role is to modulate the soil environment, which in turn acts as a systemic signal. For instance, the improved soil moisture and structure provided by biochar lead to a significant downregulation of ABA biosynthesis genes such as NCED3 (9-cis-epoxycarotenoid dioxygenase) and ABA-responsive genes like RD29B and RAB18 [120].

This indicates that the plant’s root system perceives a lower level of hydro-stress, reducing the need to activate costly drought-avoidance mechanisms. Concurrently, the more favorable growing conditions trigger an upregulation of genes central to growth and carbon assimilation, including RBCS (encoding the small subunit of RuBisCo) and LHCB (encoding light-harvesting complex proteins), directly linking the amendment to observed improvements in photosynthesis. Crucially, biochar can function as an elicitor of specific defense priming. Research in arabidopsis (*Arabidopsis thaliana*) has demonstrated that biochar amendment induces a systemic response via the jasmonic acid (JA) signaling pathway, upregulating JA-responsive genes like VSP2 (Vegetative Storage Protein 2) and LOX2 (Lipoxygenase 2). This priming prepares the plant’s immune system for a more robust response to subsequent biotic and abiotic challenges, including drought [99,121]. The molecular dialogue initiated by PGPR is more direct and targeted. PGPR that produce auxins, such as IAA, directly influence the plant’s auxin signaling network. This leads to the transcriptional activation of auxin-responsive genes, including those in the SAUR (Small Auxin Up RNA) and GH3 (Gretchen Hagen 3) families, which promote cell wall loosening and division, thereby driving the development of lateral roots and root hairs. This expanded root architecture, a key morphological adaptation to drought, is thus a direct result of bacterial-induced gene expression [122].

Furthermore, PGPR are master manipulators of the plant’s hormonal crosstalk. Through analogous process to Induced Systemic Resistance (ISR), but conferring abiotic stress tolerance (Induced Systemic Tolerance, IST), PGPR prime the JA and ethylene backbone of the plant’s defense circuitry [123]. This priming involves the upregulation of receptors and early signaling components, ensuring that when drought stress occurs, the activation of defense genes like PDF1.2 (Plant Defensin 1.2) is faster and stronger. A cornerstone of PGPR action is the enzyme 1-aminocyclopropane-1-carboxylate (ACC) deaminase. This enzyme hydrolyzes the ethylene precursor ACC into ammonia and α-ketobutyrate in the rhizosphere. By depleting the substrate for ethylene synthesis, ACC deaminase-producing bacteria effectively suppress the stress-induced ethylene burst [124]. This results in the downregulation of ethylene-responsive transcription factors (ERFs) that typically activate senescence-related and growth-inhibiting genes, thereby allowing the plant to sustain root and shoot growth under drought conditions that would otherwise trigger growth arrest. In an Italian study, the co-application of selected microbial consortia and biochar to wheat and maize systems yielded multiple benefits: (i) increased shoot and root biomass, (ii) enhanced biodiversity of the native rhizospheric microbial community, and (iii) transcriptional evidence of beneficial crosstalk between the microbiota and host plants [125]. The synergy in a combined biochar–PGPR treatment arises from the amplification and integration of these individual transcriptional signals. Biochar acts as a “microbial incubator,” its porous structure providing a protective niche that enhances the survival, colonization, and metabolic activity of PGPR. This enhanced microbial presence translates into a stronger and more sustained molecular signal from the PGPR to the plant. The improved soil moisture from biochar ensures that the roots remain active and capable of perceiving and responding to these bacterial signals. The convergence of the biochar-elicited JA priming and the PGPR-mediated JA/ethylene and auxin signaling creates a uniquely resilient transcriptional state. This synergistic interaction likely fine-tunes the expression of master regulatory transcription factors such as the DREB/CBF (Dehydration-Responsive Element-Binding protein) proteins, which control large suites of osmotic stress-responsive genes, and NAC and WRKY factors that govern senescence and defense [126]. The result is an optimized transcriptome where stress-responsive genes are activated with high efficiency without overwhelming the plant’s metabolic budget, while growth-promoting genes remain expressed. This sophisticated molecular orchestration, mediated by the complementary actions of biochar and PGPR, allows the plant to achieve a level of drought tolerance that is fundamentally greater than the sum of the effects induced by each amendment alone.

**Figure 4:**
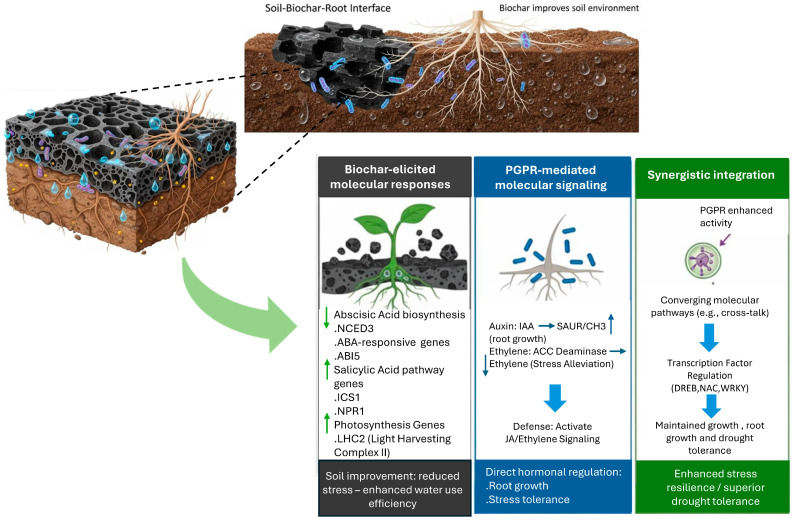
Integrated signaling and transcriptional regulation in the biochar–PGPR synergy. ABA: Abscisic Acid, ACC: 1-Aminocyclopropane-1-carboxylic acid, IAA: Indole-3-acetic acid, JA: Jasmonic Acid, ABI5: Abscisic Acid-Insensitive 5, DREB: Drought-Responsive Element-Binding protein, ICS1: Isochorismate Synthase 1, NCED3: 9-cis-Epoxycarotenoid Dioxygenase3, SAUR: Small Auxin Up RNA, NAC: NAM, ATAF, and CUC transcription factor family, WRKY: WRKY transcription factor family, LHCII: Light-Harvesting Complex II, NPR1: Non-expressor of Pathogenesis-Related Genes 1. Directional arrows denote increase (↑) or decrease (↓).

To sum up, the combined action of biochar and PGPR at the soil–root interface creates a powerful defense system for plants. Figure 4 presents the integrated signaling and transcriptional network responsible for this synergy, depicting how the molecular responses elicited by biochar and the signaling mediated by PGPR converge to achieve enhanced stress tolerance and superior drought resilience.

## 9. Conclusions and Future Perspectives

This review synthesizes compelling evidence that the co-application of biochar and PGPR represents a powerful, synergistic strategy for enhancing crop drought tolerance. As detailed, biochar serves as a foundational soil amendment, directly mitigating drought stress by improving the soil’s physical structure, enhancing its water-holding capacity, and increasing nutrient availability. This ameliorated rhizosphere environment is the first signal in a cascade that reprograms the plant’s stress response. Concurrently, PGPR act as dynamic biological agents that directly influence plant physiology by modulating phytohormone levels, producing osmolytes and exopolysaccharides, and solubilizing essential nutrients. The synergy between the two is profound: biochar’s porous structure provides an ideal protective habitat and carbon source that enhances the survival, colonization, and efficacy of PGPR, effectively amplifying their beneficial effects.

The most significant insight explored in this review is that these physiological and soil-level benefits are correlated with, and likely orchestrated by, a profound reprogramming of the plant’s transcriptome. The combination of biochar and PGPR shifts the plant’s gene expression profile from a stressed state to a resilient one. This is characterized by the downregulation of ABA-biosynthesis and responsive genes (e.g., NCED3, RD29B), as demonstrated in studies where the biochar–PGPR combination reduced ABA levels and downregulated dehydrin genes, the suppression of ethylene-driven senescence via ACC deaminase activity, and the potential priming of JA-dependent defense pathways. Concurrently, it promotes growth through the upregulation of genes involved in photosynthesis (RBCS, LHCB), auxin-mediated root development (SAUR, GH3), and the activation of master stress-regulatory transcription factors like DREB, NAC, and WRKY. The reprogrammed transcriptional network thus enables the plant to sustain growth and productivity during drought, mitigating what would otherwise be severe yield losses.

Despite the robust evidence from controlled studies, a significant translation gap exists between laboratory findings and widespread field application. To bridge this gap, future research must pivot toward strategic, application-oriented directions. Furthermore, the development of context-tailored and practical formulations is essential; this involves creating specific “biochar-PGPR consortia” matched to soil types and optimizing application protocols, including using biochar as a protective carrier for the bacteria. Concurrently, rigorous assessment of long-term agronomic and economic viability through multi-year field trials is indispensable. To enhance practicality and scalability, strategies such as using local agricultural waste for low-cost biochar production, conducting farmer-participatory on-farm trials, and exploring eligibility for carbon credit markets are crucial for making this approach economically attractive for land managers. In conclusion, the biochar–PGPR partnership moves beyond being a simple soil amendment to become a holistic management system. By simultaneously engineering the soil environment and the plant’s internal stress response network, this approach offers a sustainable and powerful tool to enhance global food security.

## Figures and Tables

**Table 1:** Effects of PGPR with various types of biochar on reducing the effects of drought on plants.

PGPR	Biochar Feedstock and Rate	Plant	Results for Drought-Stressed Plants	References
Biozote-Max, a PGPR based biofertilizer developed in Pakistan	Cotton (*Gossypium* sp.) straw 1% and 2% *w*/*w* and soil	Peanut (*Arachis hypogea*)	2% biochar + PGPR reduced leaf superoxide dismutase (SOD) by 27% in a pot experiment	[104]
*Staphylococcus* sp.	Mulburry (*Morus alba*) wood 25% *v*/*v*	Canola (*Brassica napus*)	Biochar + PGPR increased nutrient uptake, leaf RWC, sugars, proteins, flavonoids, phenolic compounds, and enzymes (peroxidase (POD), SOD, glutathione reductase (GR), and dehydroascorbate reductase (DHAR)) in drought-stressed plants in a pot experiment.	[105]
*Bacillus paramycoides* *B. thuringiensis* *B. tropicus*	*Dalbergia sissoo* wood 1% *w*/*w* and soil	Wheat (*Triticum aestivum*)	Biochar + PGPRs significantly increased crop yield (100-grain weight, grain yield), and soil quality (increased available P and microbial biomass) in drought-stressed plants in a pot experiment.	[63]
*Paraburkholderia phytofirmans* *Bacillus* sp.	Cotton stem 1% *w*/*w* and soil	Soybean (*Glycine max*)	Biochar + *P. phytofirmans* increased photosynthesis and antioxidant enzymatic activities, and improved grain yield in drought-stressed plants in a pot experiment.	[106]
*Pseudomonas fluorescens*	Mistletoe (*Loranthus europaeus*) 10–30 g kg^−1^ soil	Oak (*Quercus brantii*)	Lower additions of Biochar + PGPR enhanced shoot growth of drought-stressed seedlings in a pot experiment.	[107]
*Bacillus amyloliquefaciens*	Timber-waste 15–30 mg kg^−1^ soil	Wheat	Biochar + PGPR increased growth and yield: grain yield (36%), 100-grain weight (59%), straw yield (50%); and chlorophyll a, chlorophyll b, grain N and P by 114%, 123%, 58%, and 18%, respectively, in a plot experiment.	[10]
*Pseudomonas* sp.	Mulberry wood	Canola	Biochar + PGPR increased growth traits (emergence energy, leaf area), water content metrics (RWC, moisture content), nutrient levels (N, P, K, Mg, Ca); and regulated stress by reducing osmolytes (glycine, betaine, sugar) and boosting the antioxidant system (POD, SOD, GR enzymes, phenolics, flavonoids), in a plot experiment.	[108]
*Bacillus pseudomycoides* ARN7	Peanut shell 5% *w*/*w*	Maize (*Zea mays*)	Biochar + PGPR helped mitigate combined heavy metal (Ni, Zn) and drought stress. They significantly improved plant growth, total chlorophyll, proteins, and relative water contents. They enhanced the antioxidant enzyme system (SOD, ascorbate peroxidase (APX), and catalase (CAT)) while reducing stress markers (electrolyte leakage, MDA, proline) in a pot experiment.	[109]
*Serratia odorifera*	Santa-Maria feverfew (*Parthenium hysterophorus*) 2.5% *w*/*w*	Barley (*Hordeum vulgare*)	Biochar + PGPR increased shoot length (37%), fresh biomass (52%), dry biomass (62%), seed germination (40%), chlorophyll a (28%) and b (35%), antioxidant enzyme activity (POD, CAT, SOD), and improved soil N, K, P, and EL by 85%, 33%, 52%, and 58%, respectively, in a plot experiment.	[110]
*Bacillus subtilis* *B. tequilensis*	Wheat straw, wood chips, or rice husk 1% *w*/*w*	Wheat	Biochar + PGPR increased root length (up to 70%) and shoot length (up to 82%), grains per spike (137–182%). total chlorophyll (477%) and carotenoid (423%). Key physiological indicators (membrane stability, relative water content, proline, sugar) were significantly enhanced in a pot experiment.	[111]
*Pseudomonas fluorescens*	Pine wood biochar 2% *w*/*w*	Cucumber (*Cucumis sativus*)	Biochar + PGPR promoted shoot length (88%), shoot biomass (77%), root length (89%), and root biomass (74%) in a plot experiment.	[112]
*Bacillus siamensis*	Olive prunings 3% *w*/*w*	Olive (*Olea europaea*)	Biochar + PGPR increased shoot biomass under both well-watered and water-stressed conditions. They reduced stress indicators ABA, H_2_O_2_, MDA) and downregulated key stress-related genes (dehydrins, aquaporins), indicating improved water status and stress tolerance in a pot experiment.	[113]
*Cellulomonas pakistanensis* NCCP11 *Sphingobacterium pakistanensis* NCCP246	Mulberry wood residue 5% *w*/*w*	Faba bean (*Vicia faba*)	Biochar + PGPR boosted proline (77%), glycine betaine (107%), soluble sugar (83%), and total protein (89%); enhanced antioxidant enzymes (e.g., peroxidase by 81%) and reduced stress markers MDA (54%) and H_2_O_2_ (47%); and increased phenolic compounds and flavonoids by over 50% in a pot experiment	[114]
*Pseudomonas fluorescens*	Maize cob 0.75% *w*/*w* and soil	Maize	Biochar + PGPR + Gibberellic Acid (GA_3_) increased plant dry/fresh weight, shoot/root length, protein and chlorophyll a, b, contents, in a pot experiment.	[115]
*Paenibacillus lentimorbus* B-30488	Maize stalk 5% *w*/*w*	Chickpea (*Cicer arietinum*)	Biochar + PGPR improved soil physical and chemical properties, and soil microbial diversity); increased chickpea growth, improved root architecture, modulated key phytohormones (Indole acetic acid (IAA), cytokinin (CK), jasmonic acid (JA), and salicylic acid (SA)) and upregulated the expression of stress-related genes (glutathione S-transferase (GST), APX, CAT and ACC oxidase (ACO)) to maintain cellular homeostasis in a pot experiment.	[92]
*Penicillium citrinum*	Grass 3.0% *w*/*w*	Tomato (*Solanum lycopersicum*)	PGPR-loaded biochar and foliar IAA increased root length, shoot height, plant biomass chlorophyll, proline, and antioxidant enzymes (SOD, peroxidase, CAT), and reduced the stress markers H_2_O_2_ and MDA in drought-stressed plants in a pot experiment.	[116]
*Planomicrobium chinense* *Pseudomonas putida*	Plant leaves 5 g kg^−1^	Soybean	Biochar + PGPR improved plant osmoregulant status and soil nutrient retention in a pot experiment.	[117]
*Serratia odorifera*	Algae 4% *w*/*w*	Maize	Biochar + PGPR significantly enhanced root biomass and length under severe drought (50% field capacity) in a pot experiment, demonstrating a several-fold increase compared to the non-amended control. The co-application also improved net photosynthetic rate, plant nutrient content (N, P, K), and key soil fertility parameters.	[118]

## Data Availability

No new data were created or analyzed in this study.
